# Three Decades Investigating Humor and Laughter: An Interview With Professor Rod Martin

**DOI:** 10.5964/ejop.v12i3.1119

**Published:** 2016-08-19

**Authors:** Rod Martin, Nicholas A. Kuiper

**Affiliations:** a Department of Psychology, Westminster Hall, Western University, London, Ontario, Canada

**Keywords:** humor, personality, stress, humor styles

## Abstract

Since the start of the 21st century, the investigation of various psychological aspects of humor and laughter has become an increasingly prominent topic of research. This growth can be attributed, in no small part, to the pioneering and creative work on humor and laughter conducted by Professor Rod Martin. Dr. Martin’s research interests in humor and laughter began in the early 1980s and continued throughout his 32 year long career as a professor of clinical psychology at the University of Western Ontario. During this time, Dr. Martin published numerous scholarly articles, chapters, and books on psychological aspects of humor and laughter. Professor Martin has just retired in July 2016, and in the present interview he recounts a number of research highlights of his illustrious career. Dr. Martin’s earliest influential work, conducted while he was still in graduate school, stemmed from an individual difference perspective that focused on the beneficial effects of sense of humor on psychological well-being. This research focus remained evident in many of Professor Martin’s subsequent investigations, but became increasingly refined as he developed several measures of different components of sense of humor, including both adaptive and maladaptive humor styles. In this interview, Dr. Martin describes the conceptualization, development and use of the Humor Styles Questionnaire, along with suggestions for future research and development. In doing so, he also discusses the three main components of humor (i.e., cognitive, emotional and interpersonal), as well as the distinctions and similarities between humor and laughter. Further highlights of this interview include Professor Martin’s comments on such diverse issues as the genetic versus environmental loadings for sense of humor, the multifaceted nature of the construct of humor, and the possible limitations of teaching individuals to use humor in a beneficial manner to cope with stress and enhance their social and interpersonal relationships.

**Nick Kuiper:** I would like to thank you for agreeing to be interviewed for this special humor issue of *Europe’s Journal of Psychology (EJOP)* that honors your many contributions to the psychological investigation of humor and laughter. Over the past 30 years or so you have published numerous scholarly articles, chapters and books on various topics pertaining to humor and laughter. Your creative approach, combined with a rigorous theoretical-empirical orientation to your research, has resulted in a very strong positive influence on the field. Psychological investigations of humor and laughter have increased dramatically over the past decade, with the number of published research studies soaring. Many different facets of humor and laughter have been explored, with several of these being represented in the articles presented in this special humor issue of EJOP. Taken together, these contributions further mark the importance of utilizing a rigorous theoretical-empirical approach to increase our understanding of the various psychological aspects of humor and laughter.

Perhaps we could begin this interview by going back to the start. What was it that first piqued your interest in examining humor and laughter from a psychological perspective?

[Contrib contnr1]**Rod Martin:** Soon after I began as a graduate student in the clinical psychology program at the University of Waterloo in the Fall of 1979, I began talking with my research adviser, Herb Lefcourt, about possible topics for my Master’s thesis. There had previously been a lot of research showing the adverse effects of life stress on emotional and physical health, and Lefcourt was interested in looking at personality traits that might potentially moderate these stress effects. Lefcourt was really an early proponent of positive psychology before that label became popular. He was especially interested in the factors that make some people particularly healthy and resilient, rather than focusing on mental disturbance and emotional distress.

My research advisor had become quite well-known for his earlier research on locus of control, and at that time he was conducting some studies on the potential stress-moderating effects of this personality dimension. So we began talking about what other traits or characteristics might also help people to weather stress and adversity without becoming overly distressed or ill, and we started thinking about sense of humor as an interesting research topic. There has long been a great deal of popular lore about the health benefits of a good sense of humor, but very little scientific research had been done on it. In fact, this was right around the time that Norman Cousins came out with his book *Anatomy of an Illness* (Cousins, 1976), in which he described how he supposedly used laughter to cure himself of a life-threatening disease. So this was about to become quite a hot topic. Looking back, I think I was fortunate to get into this line of research just at that time.

**Nick Kuiper:** How did this interest in humor and laughter translate into research studies?

**Rod Martin:** Given the individual differences approach that Herb Lefcourt took in his research (and which I have generally continued to follow), we first had to find a reliable and valid way of measuring people’s sense of humor. So I spent a couple of months delving into the scholarly literature looking at how earlier researchers had approached this topic. I quickly discovered that the concept of sense of humor is much more complex and multifaceted than I had thought, and there weren’t any well-established measures that seemed appropriate for our purpose. Most of the research on humor up till then had focused on humor appreciation, which involves asking participants to rate the funniness of different types of jokes, cartoons, and other humorous materials, and then looking at how these funniness ratings correlate with other characteristics of the individuals. This is a valid approach for some purposes, but I didn’t think it got at the aspects of humor that were relevant to what I was interested in. Some people might find lots of jokes amusing and enjoyable, but that doesn’t necessarily mean that they *produce* humor, or that they are able to maintain a humorous outlook particularly during times of stress. So I started thinking about other ways of conceptualizing and measuring sense of humor, particularly using self-report scales. The only published self-report measure of sense of humor I could find was the one Sven Svebak had recently developed in Norway, which seemed quite interesting but had not yet been used much in research and hadn’t really been validated.

[Contrib contnr2]One big concern we had at that time was that there might be a large social desirability bias in a self-report humor measure: a sense of humor is so positively valued that people might not be willing to admit that they don’t have one, and might not give valid answers on scales. I thought we might be able to get around this if we had participants recall past experiences of being in a variety of stressful and non-stressful life situations and asked them to report how much they would typically laugh in each situation. This led to the development of the Situational Humor Response Questionnaire, which became the main focus of my Master’s thesis. I also created the shorter Coping Humor Scale, which more directly asked participants to rate the degree to which they use humor to cope with stress in their lives (see Martin, 1996 for an overview of research using these two humor scales).

Over the five years of working with Lefcourt on my Master’s and Ph.D., we carried out a number of studies, first looking at the reliability and validity of these measures along with Svebak’s scale, and then examining stress-moderating effects using life events scales and self-report measures of positive and negative moods. We also conducted some experimental studies in which we had participants watch a very stressful and rather gruesome movie called *Subincision*, under either humorous or non-humorous conditions, and assessed their level of emotional distress via behavioral observation and self-report. Herb had quite an infectious sense of humor himself, and I remember having a lot of laughs with him and his other graduate students while doing that research. Fortunately, we got some rather nice results from those studies, and we published them in some journal articles (e.g., Martin & Lefcourt, 1983) and later in our book, *Humor and life stress: Antidote to adversity* (Lefcourt & Martin, 1986).

**Nick Kuiper:** What do you view as the most significant findings that emerged from this early work on sense of humor as a strategy for coping with stress?

**Rod Martin:** Those early studies focused on a stress-moderation paradigm, and did produce some evidence that individual differences in sense of humor moderate the association between stressful life events and negative moods (Martin & Lefcourt, 1983). In other words, individuals with higher scores on certain humor measures showed a weaker correlation between life stressors and distressed moods than did those with lower humor scores. These findings gave support to the idea that people who have more of a sense of humor are better able to cope with stress and therefore are less adversely affected by it. Those early studies helped to stimulate more interest and research on this topic and were also picked up by the media, contributing to the development of the “humor and health movement” in the 1980’s and 90’s.

**Nick Kuiper**: How do you see your early work addressing the broader issue of humor’s role in psychological well-being?

**Rod Martin:** After I was hired as a faculty member at the University of Western Ontario in 1984, I started collaborating with you, as well as some of my graduate students, on further studies on humor and stress. My first PhD student, James Dobbin, was interested in the effects of stress on physical health, and we ran some studies looking at various components of the immune system (Martin & Dobbin, 1988). Among other things, we were able to replicate the earlier stress-moderator findings using immunoglobulin A as the outcome variable, thus extending the earlier findings to physical health as well as emotional well-being. In the research with you, we explored some possible mechanisms of these stress-moderating effects. For example, we looked at how the humor measures related to cognitive appraisals of stress, and found that people with higher humor scores tended to perceive potentially stressful events as more of a challenge, whereas those with lower humor saw them more as a threat (Kuiper, Martin, & Olinger, 1993). This gave support to the idea that the benefits of a sense of humor for coping may be partly due to the way it changes the individual’s appraisals of stressors. In other studies we found that those with high humor scores tend to have more stable self-concepts over time, suggesting another possible benefit of humor for coping (Kuiper & Martin, 1993).

Later we ran a number of studies looking at the correlations between the sense of humor scales and a variety of measures of psychological well-being, including positive and negative moods, self-esteem, optimism, mastery, purpose in life, and so on (Kuiper & Martin, 1998). Surprisingly, we found that many of these well-being variables were unrelated or only weakly related to the humor scales. This got me thinking that our approach to measuring individual differences in humor may have been a little too simplistic. I started thinking more about ways that humor could be detrimental as well as beneficial for well-being, which led eventually to the development of the Humor Styles Questionnaire (Martin, Puhlik-Doris, Larsen, Gray, & Weir, 2003).

**Nick Kuiper**: Over your career you have devoted considerable energy to measuring a variety of individual differences in humor and laughter. Some of your earliest work, for example, developed both the Coping Humor Scale (CHS) and the Situational Humor Response Questionnaire (SHRQ). What do you see as the primary theoretical reasons for taking this individual differences approach to humor and laughter?

**Rod Martin:** I gravitated toward the individual differences approach mainly because that was the approach I learned in graduate school, so it has always been most familiar to me and consistent with the way I think about psychology generally. This also seems most consistent with the popular concept of “sense of humor,” which is generally viewed as a fairly stable trait or personality dimension. The major limitation of this approach, though, is that it lends itself most readily to correlational research, which has the drawback of not being able to demonstrate causal relationships between variables. If we find a correlation between a particular humor measure and some aspect of well-being, we don’t know whether the humor actually causes the well-being. An alternative approach would be the sorts of experimental methods taken by social psychologists, and certainly I see that type of research as also being very important in the study of humor and well-being. A drawback of the experimental approach, in my view, is that it can often be somewhat artificial, whereas the individual differences approach seems to have more external validity. So we need both approaches to offset each of their limitations.

**Nick Kuiper**: Although humor and laughter have been examined in many studies, these constructs can sometimes still remain a bit elusive. In this regard, how might we best define humor from a psychological perspective?

**Rod Martin:** I view humor as quite a broad and multifaceted psychological phenomenon that encompasses several components (Martin, 2007; Martin, 2016). The first is the *cognitive* aspect, namely the perception of incongruity, which has also been referred to as “bisociation” or “cognitive synergy.” It seems to involve the simultaneous activation of two or more incompatible interpretations of a situation in the mind. It also tends to be associated with a playful, non-serious frame of mind and some degree of diminishment, in which things are viewed as being less important or admirable than they usually are. These cognitive elements are what make something “funny.”

Second, there is the *emotional* component. The cognitive processes activate a unique emotional response, which I refer to as “mirth.” In the English language, this word “mirth” has a long lineage and seems to be perfect as a technical term for this emotional aspect of humor. Mirth is related to joy, but is somewhat different because of the element of “funniness” involved. It is accompanied by activation of the pleasure circuits in the limbic system as well as various autonomic and endocrine responses, and is what makes humor so enjoyable.

Third, there is the *social or interpersonal* aspect. I see humor as being fundamentally a social activity. We are much more likely to laugh with other people than when alone, and most humor arises in response to the behavior of other people or human-like traits in non-human animals. From an evolutionary perspective, I think humor evolved as a mechanism for enhancing group cohesion.

The final component is *laughter*, which I see as a hard-wired nonverbal expression or communication of the emotion of mirth. Laughter occurs also in other primates, so it has a long evolutionary history going back long before we evolved language and other higher cognitive abilities. So laughter is the way we let others know we are experiencing mirth, and it also has the effect of eliciting this emotion in the listener. That’s why laughter is so contagious. Strong laughter can also intensify and amplify the emotion of mirth. Usually this happens when people are in small groups, and they engage in intense bouts of laughter that are very enjoyable and create strong feelings of group cohesion.

Some theorists might define humor more narrowly, focusing only on the cognitive aspect, for example. But I think humor should be defined broadly enough to include the constellation of all these elements. In any one instance of humor, one or another of these elements might predominate. For example, in more cerebral types of wit the cognitive element might be primary, with very little mirth or laughter. At other times, laughter and mirth might predominate and the cognitive incongruity component may be minimal. Sometimes people experience mirth and even some laughter when alone, so the social element may be lacking, but usually this occurs in a “pseudo-social” situation such as watching a comedy show on TV or remembering an amusing incident that involved other people.

Some researchers see laughter as something quite distinct from humor, and argue that it frequently occurs as a sort of social signal of friendliness that has nothing to do with humor (e.g., Provine, 2000). However, the research evidence for that view is very limited, and I’m not convinced by it. I would draw the boundary of humor broadly enough to include most instances of social laughter. Even though people may laugh when there is very little cognitive incongruity present, I think the playfulness and diminishment aspects are typically still occurring, and certainly the mirth and social dimension.

**Nick Kuiper:** To what extent do you think contemporary researchers are cognizant of the main distinctions between humor and laughter? What would you see as the most profitable directions for future research in this area?

**Rod Martin:** I think some contemporary researchers are actually exaggerating the distinctions between humor and laughter. I agree that laughter sometimes occurs outside of humor, but that may be an anomaly. In general, I see laughter as one component of the broader constellation of phenomena of humor. However, I think it can be worthwhile to study each of these components individually. For example, psycholinguistic studies investigating cognitive aspects of humor can focus on the essential elements involved in the perception of incongruity and what makes something funny, without being concerned with the emotion of mirth or laughter or the social dimension. Other researchers may be more interested in focusing on the emotional component and the neurological and physiological aspects. Others may focus on the social dimension or on laughter. In fact, I think this kind of narrowing in on various sub-components might be the best way to make progress in understanding the broader phenomenon of humor. Even for those of us who are particularly interested in psychological and physical health aspects of humor, it might be beneficial to focus on particular dimensions individually and see how they relate to various aspects of health and well-being. For example, the cognitive component of humor may be particularly important for coping with stress, whereas the emotional aspect may be particularly relevant for physiological health.

**Nick Kuiper:** More recently you have developed an assessment instrument to measure four different humor styles, namely, the Humor Styles Questionnaire (HSQ). This scale provides a measure of individual differences in affiliative humor, self-enhancing humor, aggressive humor and self-defeating humor. Could you please take us through the thinking and process that led to the development and validation of this scale?

**Rod Martin**: In the late 1990’s I started thinking more about the idea that the relevance of humor to health and well-being may have more to do with the way people *use* humor than the overall degree to which they have a “sense of humor.” Some people can be very funny and comical without necessarily being particularly healthy from a psychological perspective. We only need to look at comedians like Chris Farley and John Belushi for examples of this. I went back to the writings of some earlier psychologists such as Abraham Maslow and Gordon Allport, and even Sigmund Freud, and looked at how they distinguished between healthy and unhealthy forms of humor. This led me to the idea of looking at the psychosocial functions of humor in everyday life, some of which may be beneficial for well-being while others may be detrimental.

I worked particularly with one of my graduate students, Patricia Puhlik-Doris, on the development of the Humor Styles Questionnaire, and both her Masters and Ph.D. focused on this measure. In coming up with the four dimensions, we were influenced by the research on agency and communion as two primary, orthogonal dimensions underlying interpersonal traits and behavior. Agency has to do with individual autonomy and control, whereas communion relates to social connectedness. So we saw self-enhancing and aggressive humor as being on the agency dimension – one healthy and the other unhealthy – whereas affiliative and self-defeating humor were healthy and unhealthy forms on the communion dimension.

In developing the HSQ, we quickly found out how difficult it is to assess these different functions of humor using a self-report format. I don’t think most people are consciously aware of the implicit goals and psychological effects that their humor may have at any given time. They’re only aware that they’re laughing because something seems funny to them. Similarly, I don’t think most people use humor in a strategic way to achieve particular goals. We don’t say, “I’m going to say something funny now in order to cope with this stressful situation or to make that person look like an idiot.” Instead, humor tends to occur quite spontaneously most of the time, arising out of unconscious processes. Nonetheless, I think we can see that humor serves various functions by looking at the patterns of consequences over time.

In our first attempts at creating items for the HSQ we tried to ask research participants directly about the degree to which they engaged in humor for various purposes. But people had a hard time answering these questions, and we were unable to get any reliability. So then we worked on items that get at the functions of humor more indirectly, asking more about the typical context and consequences of their use of humor. Over a couple of years we went through several revisions of the scales, using a number of different subject samples, before arriving at a final measure with four scales showing good reliability, discriminant validity, and a consistently stable factor structure (Martin et al., 2003; Martin, 2007).

**Nick Kuiper:** The HSQ has been a phenomenally successful assessment instrument, with over 125 published studies using the measure and more than 500 citations. Why do you think the HSQ has been so broadly endorsed and employed in contemporary humor research? What are its main advantages for research on humor?

**Rod Martin:** It certainly has been gratifying to me to see how widely used the HSQ has become. It has now been translated into over 30 languages, and I frequently get emails from researchers all over the world asking about it. I think it has gained such wide acceptance in part because it did turn out to be more strongly predictive of various aspects of psychosocial well-being than the earlier humor measures. The four scales do seem to have quite distinct patterns of correlations, both positive and negative, with a range of variables involving well-being, personal relationships, and personality more generally. So it does seem to have been quite successful in doing what we set out to do with it. The fact that we put so much care and effort into developing the HSQ also likely helped, both with the strength of the results, and with its perceived usefulness for further research. Also, I think the HSQ came along just at a time when research on humor was starting to become more main-stream in psychology. Previously, I think humor was seen as a topic that was perhaps not “serious” enough for respectable research. The positive psychology movement was probably a factor in making topics like this more respectable.

Also, some brain imaging studies came out around that time, showing that particular areas of the brain are activated in response to humor. There seems to be an odd perception that if something can be seen in the brain, it is more real and scientifically valid. As more researchers started getting interested in looking at humor, the HSQ happened to be available as a reliable and reasonably well validated measure, so they naturally started using it, giving it further momentum.

**Nick Kuiper:** What are some other ways that you see the HSQ being used in the future?

**Rod Martin:** In recent years, my students and I have been developing versions of the HSQ for different purposes. For example, we have peer-report formats and also versions specific to particular relationships such as friendships and dating relationships. This has led to some interesting findings about the role of humor styles in different types of relationships. Also, we’ve been developing versions for assessing humor experiences over shorter time frames rather than using it as a trait measure. We’ve used these in a number of daily diary studies, looking at the way within-person changes in the frequency of the different styles of humor from day to day are associated with corresponding changes in individuals’ positive and negative moods, relationship satisfaction, and so on. This has led to some interesting findings of different patterns of results within individuals over time as compared to the cross-sectional findings. For example, one of my recent graduate students, Kim Edwards, found that, for people who don’t engage in self-defeating humor very often overall, this style of humor actually tends to be *positively* associated with psychological well-being on a day-to-day basis (Edwards, 2013; Edwards & Martin, 2014). However, for people who use it a lot, it is *negatively* related to well-being. So this research helps to tease out some more fine-grained nuances of the humor styles.

Another avenue of research that I’ve been involved in with my students and my colleague Lorne Campbell is to develop an observational coding system based on the HSQ framework. We’ve used this system to rate the humor that occurs naturalistically in dating couples while they’re engaged in conversations about conflict-related issues in their relationships, and also in friendship dyads discussing stressful experiences in their lives (Campbell, Martin, & Ward, 2008). We found that these humor style ratings were predictive of various outcomes such as feelings of satisfaction and perceptions of problem resolution following the conversations. By observing humor in “real time” like this, we can start to see how different styles of humor may lead to various outcomes. I think there is still a lot of potential for further research using these different kinds of methodologies based on the HSQ framework.

**Nick Kuiper**: In the HSQ two of the humor styles are generally considered to be adaptive (affiliative & self-enhancing) whereas two are generally considered to be maladaptive (aggressive & self-defeating). How hard and fast do you see this distinction to be? Could you envision instances were maladaptive humor use may actually prove to be facilitative (e.g., a small amount of self-defeating or aggressive humor used in the right context)? How might researchers develop a means of examining this issue? What is necessary for investigators to consider in order to come to a more complete understanding of the function of humor from an adaptive versus maladaptive perspective?

**Rod Martin:** From the outset, we saw the different humor styles as having rather fuzzy boundaries. Some styles of humor may be benign or even beneficial when used sparingly, but detrimental when used excessively. The diary study findings with self-defeating humor that I mentioned previously are a good example of that. As long as you don’t use it too much, it may actually be beneficial. Also, I think the differences between healthy and unhealthy forms of humor can be very subtle. For example, I think there is a difference between *self-defeating* humor and *self-deprecating* humor. Self-deprecating humor is a healthy form of humor in which you don’t take yourself too seriously and are able to laugh at your own mistakes in a self-accepting way. I think this comes out of healthy self-esteem. In contrast, self-defeating humor arises from low self-esteem, and involves excessively self-disparaging humor that is used to ingratiate oneself with others. However, it’s often difficult to distinguish between the two in any single instance. Similarly, friendly types of teasing can be a form of healthy affiliative humor, whereas more destructive teasing is part of aggressive humor. Again, it can sometimes be difficult to distinguish between them. Also, it’s important to recognize that we conceptualized the four humor styles as being relatively independent of each other, meaning that people can be high or low on more than one of them. Some people who are high on affiliative humor also engage in a lot of aggressive humor, and in fact the two styles tend to be weakly positively correlated, at least in western cultures.

I certainly don’t see the HSQ as the final word on healthy and unhealthy forms of humor, and I have no doubt that it will eventually be replaced by something else, as research progresses. I think it has been useful for identifying some broadly-defined functions of humor and showing different overall patterns of associations with aspects of well-being. But I think future research will need to become more fine-grained, breaking these humor styles into smaller components, and also looking at them in various combinations. There may also be other relevant humor styles that are not currently included in the HSQ that will be identified by future researchers. It will also be useful to pay more attention to the interpersonal context of the humor. For example, aggressive humor is likely to be less detrimental when directed towards a member of an out-group than when directed at someone within the in-group. For this kind of research, we may need to move away from the trait approach and self-report scales and do more observational and experimental studies.

**Nick Kuiper:** When talking about humor styles, what has research indicated in terms of the relative genetic and environmental loadings for each style? What are some of the broader ramifications of these loadings? For instance, what do these loadings suggest for any attempts to alter an individual’s characteristic pattern of use for the four humor styles?

**Rod Martin:** I’ve collaborated with my colleague Tony Vernon and others on some twin studies to determine the heritability of the humor styles. The first study seemed to show that the two positive humor styles have a sizable genetic contribution, whereas the two negative styles are entirely influenced by environmental factors (Vernon, Martin, Schermer, Cherkas, & Spector, 2008). However, in subsequent studies we found that, for all four humor styles, about half of the variance can be explained by genetics and half by environmental influences (Vernon, Martin, Schermer, & Mackie, 2008). This is very similar to what researchers have found for most personality traits, such as extraversion, agreeableness, neuroticism, and so on. In fact, the twin studies also show that part of the variance in humor styles is due to the same genetic factors underlying these broader personality traits. In other words, the different humor styles can be viewed as expressions of particular personality traits, at least to some extent. Affiliative humor tends to be an extraverted style of humor, for example. I would interpret these findings as indicating that humor styles can be changed to some degree, but it would be difficult to change them a lot. Just as it’s very difficult for an introverted person to become an extravert, it would be hard for a person who is low on affiliative humor to become someone who is always telling jokes, engaging in witty banter, and making others laugh.

**Nick Kuiper:** In considering “sense of humor”, it is clear that current researchers view this construct as being multi-dimensional in nature. One prominent example, of course, is the HSQ, with two of the humor styles often thought of as being more adaptive (e.g., affiliative and self-enhancing humor) and two being more maladaptive (e.g., aggressive and self-defeating humor). In addition, the HSQ also considers the self versus other focus of the humor style being used (e.g., self-defeating versus aggressive). It is also the case, however, that sense of humor can be divided up in many other ways. Just one example distinguishes between humor appreciation (the ability to enjoy or appreciate humor in one’s environment) versus humor generation (the ability to generate humorous or witty comments in response to various interactions with one’s environment). If you were to build upon the HSQ, what other dimensions or facets of sense of humor would you consider adding?

**Rod Martin:** I certainly agree that “sense of humor” is a multifaceted concept. When we think of the different components of humor that I talked about earlier (cognitive, emotional, interpersonal, expressive), it’s clear that there are many different humor-related dimensions along which people can differ from one another. These different dimensions are not necessarily correlated with each other, and some may even be negatively correlated. Humor creation ability seems to be completely unrelated to humor appreciation, for example. So I don’t think we’ll ever be able to capture all the relevant dimensions in one measure alone.

When we developed the HSQ, we were interested in certain aspects of humor that might be particularly relevant to psychosocial health and well-being. We were not trying to create a comprehensive measure of “sense of humor.” There are lots of other dimensions, such as humor appreciation and humor creation ability that are likely not very relevant to psychological well-being. Overall, I don’t believe that someone needs to have a good sense of humor to be psychologically healthy. Some very funny people who laugh and joke a great deal have a lot of emotional disturbance and dysfunctional relationships, and some very serious, introverted people are well adjusted and have healthy personal relationships. So I don’t think I’d try to incorporate these other dimensions into the HSQ, since this was not the purpose of it.

**Nick Kuiper:** Do you see it as being possible to try and develop a very broad-based sense of humor measure that would incorporate the most important facets or dimensions of sense of humor, as described in the contemporary humor literature? What might such a measure look like?

**Rod Martin:** I think it would need to be more of a battery of tests rather than a single measure. I’m thinking of something like the Wechsler intelligence tests that have a dozen or so sub-tests, each designed to assess a different aspect of intelligence. In a comprehensive humor test battery there would need to be different sub-tests for getting at different humor dimensions, such as humor appreciation, humor creation ability, humor styles, and so on. They would also need to be different types of tests, some being maximal performance-type tests to assess humor-related abilities, others assessing typical trait-like behaviors, some involving behavioral observation or peer reports, and others using self-report methods. Instead of leading to one overall “humor IQ” score, this test battery would produce a profile of scores on a number of different dimensions, mapping out each individual’s unique combination of humor-related abilities, preferences, styles, strengths, and weaknesses.

**Nick Kuiper:** How might such a broad-based sense of humor measure be used in research to help advance our understanding of an individual difference approach to humor and laughter?

**Rod Martin:** For the most part, I think each dimension would likely need to be studied independently in relation to other variables, because I’m assuming that they’re largely independent factors. Thus, I think they would each show different patterns of relationships with other personality traits, well-being variables, abilities, and so on. There might also be some interesting interactions among different humor dimensions, such that one dimension might moderate the relationship between another dimension and some other personality trait or ability. The possibilities here are endless!

**Nick Kuiper**: Let’s talk a bit more about the ways that humor may be related to stress and personal well-being. Underlying this type of research is a major distinction between humor that an individual might be exposed to (for example, watching a comedy film) versus humor that is a personality characteristic or trait of the individual (for example, having a “good sense of humor”). Perhaps you could comment on the usefulness of this basic distinction in humor theory and research, while also highlighting some of the major findings in this domain. What might you suggest for future research directions?

**Rod Martin:** I think the distinction you’re talking about also relates to a broader distinction that I make between what I call “performance humor” versus “conversational humor.” Performance humor includes things like television sit-coms, stand-up comedy, humorous books and movies, which are mostly produced by people who make their living on humor. Conversational humor involves everyday joke-telling, humorous personal anecdotes, witty banter, irony, and other funny comments that tend to occur spontaneously in all sorts of social interactions. Performance humor is certainly an interesting topic for research, but I’ve always assumed that conversational humor is much more relevant for health and well-being, which is what I’ve been most interested in. Spending a lot of time laughing at sit-coms on TV is likely to make you *less* healthy, rather than more healthy! In my view, if there really are any emotional or physical health benefits of humor, they’re more likely to come from conversational humor.

At the same time, though, I think it can be very useful to employ comedy videos and other types of humorous materials in experimental studies to investigate psychological and physiological effects of humor. There have been quite a few studies in which participants are randomly assigned to view either humorous or non-humorous videos, in order to examine the effects of humor on mood, blood pressure, heart rate, immune system functioning, etc. These are the sorts of experimental investigations that are useful for demonstrating causal effects, as I mentioned earlier. I certainly think there’s a need for more of these kinds of studies in this area of stress and well-being, rather than relying too much on correlational research.

However, I would tend to see the comedy videos and other humorous materials in these types of experimental studies as being a sort of “stand-in” for the conversational humor that occurs in everyday life. If an experiment shows that watching a comedy video causes participants to be less adversely affected by some sort of laboratory stressor, I wouldn’t think the take-home message is that people should spend more time watching television comedy in order to cope better with stress in their lives. Instead, I’d extrapolate the findings to the kinds of humor that people can generate and enjoy in their everyday social interactions. This gets back to my earlier comment that experimental approaches can often be somewhat artificial. It would be good for experimental researchers to develop more realistic ways of manipulating humor in the laboratory besides videos, in order to increase the external validity. But greater external validity often comes with less control over the variables being manipulated. So again, there’s always a trade-off with different research approaches, and we need to use multiple approaches to triangulate on the truth.

**Nick Kuiper:** To what degree do you think it is possible to teach individuals how to use humor in a positive adaptive manner to manage stress? Similarly, can individuals also be taught to stop using maladaptive humor that is detrimental to their well-being? Along the same lines, what is your take on the current state of empirically-based research on humor-based interventions for improving psychological well-being?

**Rod Martin:** I think it’s probably more difficult to teach people to improve their ability to create humor than to teach them to use the humor they already have in a more adaptive way. If someone is not very funny to begin with, it’s very hard for them to learn to be more witty. But someone who is already constantly cracking jokes, but doing it in a maladaptive way, might be able to learn more adaptive ways of being funny.

Overall, if we’re trying to help people with psychological difficulties and disorders, I think it’s more beneficial to target broader psychological issues rather than to try to modify humor directly. This is why I’m not very keen on the idea of humor-based therapies. I think that more established approaches like cognitive-behavioral therapy can be very beneficial for helping people to modify their maladaptive cognitions, behaviors, and emotions. More healthy styles of humor are likely to come along with the resulting improvements in mental health, without necessarily needing to target them directly.

At the same time, though, I think many psychotherapists could benefit from being more aware of the functions of humor in their clients’ lives and in the client-therapist relationship, and more alert to ways in which maladaptive humor may play a role in their clients’ psychological dysfunctions. So some targeting of humor styles in therapy could be beneficial, as an adjunct to other therapeutic techniques.

On the other hand, for people who are already reasonably healthy psychologically, some sort of humor training might potentially be useful for enhancing their well-being further. I think it might be worthwhile for researchers to investigate these sorts of interventions more as a way of enhancing well-being rather than treating disorders. This is in line with a positive psychology approach, where the focus is on developing simple exercises that can enhance happiness and life satisfaction in generally healthy people. One of my recent graduate students, Kim Edwards, focused her research on the role of humor in positive psychology. As part of her PhD, she developed a humor-based intervention for enhancing well-being based on the humor styles framework, and found that it produced significantly greater improvements in positive mood compared with a no-treatment control group, but no reduction in negative moods (Edwards, 2013).

A few other studies have come out recently showing fairly promising results with some of these types of humor-training programs (e.g., Falkenberg, Buchkremer, Bartels, & Wild, 2011), but I don’t think we’re at the point yet where we can have much confidence in their effectiveness. More research is needed to identify which components of the interventions are most effective, what types of people are most likely to benefit, and to compare them with other non-humor interventions. I’ve always frowned on those who jump on what I call the “humor promotion bandwagon” and run far ahead of the research evidence, making unsubstantiated claims about benefits of humor. I think they risk doing more harm than good.

**Nick Kuiper**: Humor is also quite relevant to close interpersonal and social relationships. In your research you have conducted a number of studies that have looked at various aspects of close interpersonal and social relationships that bear on humor. Can you tell us a bit about this research and what you consider the major findings to be?

**Rod Martin:** I’ve always viewed healthy personal relationships as being an essential component of overall psychological health. I think of humans as fundamentally social animals. We evolved in small groups, and to function well we need to be able to get along well with others, both in our close relationships and in more casual interactions. There is a lot of research supporting these ideas. Also, as I said earlier, I see humor as being essentially a social phenomenon. So in studying potential benefits of humor for mental health, it was quite a natural step for me to look at interpersonal relationships in addition to emotional well-being.

Quite a lot of the research that I’ve done with my graduate students over the past 10 or 15 years has focused on relationships using the humor styles framework. We’ve looked particularly at the role of humor in dating relationships and in close friendships. A lot of this research made use of daily diary techniques to get at within-person, day-to-day changes in humor styles in relation to relationship satisfaction and related variables. Not surprisingly, these studies have shown that, on days when people engage in more positive humor styles with their partners, particularly affiliative humor, they tend to have higher levels of satisfaction in their relationships, whereas negative humor styles on a given day, particularly aggressive humor, are associated with more dissatisfaction that day (Caird & Martin, 2014). Recent research by my student Sara Caird was designed to examine potential mediators of these effects (Caird, 2015). She found that these day-to-day associations between humor styles and relationship satisfaction were mediated by changes in intimacy and positive and negative moods. In other words, when individuals engage in more adaptive forms of humor with their partners, they experience an increased sense of intimacy and more positive and less negative moods, which in turn lead to greater satisfaction with the relationship.

Other research on interpersonal relationships made use of observational methods. Another one of my graduate students, Jennie Ward, had pairs of close friends come into the lab and engage in a videotaped conversation in which one of them (the “discloser”) talked about some stressful situation that he or she was currently dealing with, while the other (the “supporter”) was to respond supportively (Ward, 2008). We then coded the humor styles of both friendship partners in each dyad, and looked at how these were related to their moods and perceptions of how helpful the conversation was. Interestingly, we found that different styles of humor were important, depending on which role the individual was playing. For the disclosers, their expressions of affiliative and self-enhancing humor were particularly related to how well they felt afterwards. On the other hand, for the supporters, the absence of aggressive humor was more important than the presence of the positive humor styles.

Another one of my recent graduate students, Dave Podnar, investigated the role of friendly teasing in relationships (Podnar, 2013). He found that even friendly teasing is not as benign as commonly thought. People who engage in a lot of this sort of kidding tend to be less well liked by others, and tend to have aggressive, non-empathic personalities.

**Nick Kuiper**: In closing, I would like to thank you once again for sharing your comments and views with us. I would also like to note that you have just recently retired this summer, and I was wondering if you would share with us some of your plans for retirement.

**Rod Martin:** I’m looking forward to having some time to relax, read, travel, and pursue other hobbies. My wife, Myra, and I now have eight grandchildren, so that’s enough to keep us busy! We also just bought a motorhome and are looking forward to doing some traveling across Canada and the United States over the next few years. We plan to take some trips overseas as well. I’m hoping also to have some time for hobbies like oil painting and woodworking. However, I also hope to have some ongoing involvement in humor research. The publisher of my book, *The Psychology of Humor* (2007), has been encouraging me to write a revised edition, so I expect to be working on that in coming months. I also hope to continue attending the annual conferences of the International Society for Humor Studies, which meets in different countries each year. Looking back over the years, I value the many good friendships I’ve made with humor scholars from various disciplines all over the world, and I hope to keep in touch with them and keep up on developments in their research.

**Nick Kuiper:** Any last words?

**Rod Martin:** I’d just like to thank you, Nick, for editing this special journal issue on humor research. It’s a real honor for me. I appreciate also the opportunities you and I have had to collaborate on research together over the years. I’ve enjoyed having you as a colleague.
